# Insomnia and Death Anxiety: A Theoretical Model with Therapeutic Implications

**DOI:** 10.3390/jcm12093250

**Published:** 2023-05-02

**Authors:** Nathaniel F. Watson

**Affiliations:** Department of Neurology, University of Washington School of Medicine, Seattle, WA 98104, USA; nwatson@uw.edu

Insomnia is common, growing in prevalence [1], often comorbid and challenging to treat [2]. As much as 50% of the adult population periodically experience insomnia, with 10% to 15% suffering chronically [3]. Whether the problem is difficulty falling or staying asleep, frequent awakenings with difficulty returning to sleep, or nonrestorative sleep, insomnia has a tremendous untoward impact on health (e.g., physiology, daytime functioning) and well-being. Insomnia costs the U.S. economy in excess of 100 billion annually, mostly due to problematic effects on workplace presenteeism/absenteeism, accident risk and healthcare utilization [4]. Because human psychophysiology concerns the way the mind and body interact, psychological dynamics contribute to insomnia causation and propagation in nearly all those experiencing the disorder [5]. 

In this Special Issue, we address numerous novel perspectives on insomnia management. These include the treatment of insomnia comorbid with anxiety and depression [6], the long-term use of insomnia treatments [7], the impact of insomnia treatment on daytime symptoms [8], the suitability of trazodone as a first-line therapy [9], and best practices for switching or deprescribing medicinal insomnia therapies [10]. Here, I present a theoretical model of how personal management of ego orientation may mitigate or reduce insomnia expression through effects on the experience of death anxiety. 

For many with insomnia, the body desires to sleep, but the mind refuses to cooperate. Intrusive negative thoughts or worries, and the negative feedback loop of concern that a lack of sleep portends catastrophic consequences for the following day’s activities, actively sabotages sleep attainment. Why, when we are comfortable, relaxed, and reposed, facing no immediate demands, with an opportunity to rejuvenate, do we promulgate these unpleasant sleep-sabotaging thoughts? Why do we not steer our consciousness towards more pleasant or near neutrally pleasant thoughts or memories to foster sleep? Why do we become negative, venturing into the inner world of anxious thought, catastrophizing sadness, worry, and paranoia? Why do we, at times, have unrealistic, inflated conceptualizations of sleep as a perfect process that results in unfulfilled expectations? Why do we choose to externalize insomnia causation and seek to place blame rather than self-determine our sleep success? What is it about the human condition that would foster such a self-sabotaging outlook when a healthy human mind can control its thoughts and choose a more positive frame of mind?

In 1637 French philosopher René Descartes published *Discourse on the Method,* where he first presented the phrase, “I think, therefore I am” [11]. However, to fall asleep is not to think. Human self-awareness is considered a defining characteristic of our species [12]. At any given moment, we understand we exist within a body of a certain age, whether healthy or ill, within a world we occupy, at a discreet moment in time. We also understand that we will not live forever, and our end can come at any moment. Death is our final destination, but the why, where, when and how are unknown. To consider death is to ponder our own annihilation—the complete destruction of our self and our ego, everything we have thought or achieved and everyone we have known or loved.

The existential reality of death fosters thanatophobia, also known as death anxiety—the fear of your own death or the process of dying [13]. This phenomenon results from psychological conflict between our self-preservation instinct and death’s capricious inevitability. The grim reality of death subconsciously ushers forth the acknowledgment of our own mortality and its associated fears [14]. Thanatophobia is a “transdiagnostic construct” via its linkage to several other medical conditions, particularly depression, anxiety, and panic disorders [15]. In this Special Issue, Morin et al. describe the tight link between insomnia, anxiety and depression and the treatment challenges therein [6]. Emerging research suggests that death anxiety impacts sleep processes, as exemplified by an association with bedtime procrastination in men [16] and death anxiety scores being positively associated with nightmare occurrence, death representation in dreams, and recurring nightmares [17]. A principal components analysis identifies death fear in sleep as a substantial contributor to the experience of death anxiety [18]. Age and sex are important factors in death anxiety, with older age women experiencing greater levels of anxiety when presented with the concept of death than other groups [19]. Correspondingly, insomnia is more prevalent in women than men and insomnia prevalence increases with age [20]. Although thanatophobia is not a formal diagnosable condition in the Diagnostic and Statistical Manual of Mental Disorders, fifth edition (DSM-5), this issue may be the nidus at the center of the fear driving much human psychopathology (e.g., anxiety, post-traumatic stress disorder, depression). Here, I posit that death anxiety tilts the balance of the human mind towards negative thought processes directly related to attempted sleep that fosters the development and perpetuation of insomnia. 

Sleep is as crucial to health and well-being as diet and exercise [21]. For most, we can prepare and eat whatever we require whenever we want, and exercise is as attainable as a walk outside. Sleep, however, is not controllable. Sleep is something that happens given the right circumstances, not something a person does per se. Actively trying to sleep can make falling asleep more challenging. When we have a basic physiological need in order to live, yet we cannot control the attainment of that need, then the integrity of our ego, our sense of self, and our very existence, is threatened, and death anxiety may emerge. Ego threat can result from a sense of decreased control over negative events. Insomnia is a negative event involving the absence of control. A threatened ego is fertile ground for the emergence of death anxiety, which, by fostering negative self-talk, anxiety, and a depressed mood, further exacerbates insomnia. Quieting the ego offers a pathway out of this negative feedback loop. 

There are four key aspects of a quiet ego: detached awareness or “mindfulness”, inclusive identity or a sense of interconnectedness with others, perspective-taking (a precursor to empathy), and growth [22]. Both mindfulness and self-empathy are important elements of cognitive behavioral therapy: a common and effective treatment for insomnia [23]. A quiet ego, most exemplified by humility, appears to buffer death anxiety. Humility involves a willingness to accept the self and life without comforting illusions and reduced levels of self-focus. Consequently, it renders the notion of mortality less threatening and less likely to evoke negative behaviors or psychological illness [24]. It also may render the sleep challenges experienced by those with insomnia less deterministic and more phenomenological. As such, a challenging night of sleep becomes just that, and not a broader concept portending the destruction of the self, and thus, prevents the issuing forth of anxiety and fear that so often propels the insomnia problem. 

Death anxiety can promulgate a “loud ego”, which, according to terror management theory, protectively motivates people to guard and promote their self-esteem and worthiness, thus supporting the belief that they play a vital role in a meaningful world [22]. Yet, humility, acceptance, and reduced self-focus are necessary to mitigate death anxiety [22]. In essence, either we build a socio-behavioral wall to protect ourselves from death anxiety, or we diffuse it with humility. Thus, when managing death anxiety, the protective wall is likely insomniogenic, while humility is likely insomniolytic. Thus, the manner in which death anxiety is managed by the ego’s orientation may contribute to insomnia development and perpetuation (Figure 1). 

How can we utilize this theoretical model to improve insomnia treatment outcomes? We should teach our patients humility in the face of problematic human psychophysiology. To be humble when approaching insomnia is to acknowledge you lack complete control over your ability to sleep, and the more you try to control it or force it, the less successful you will be in the long term. Humility is the acceptance of the insomnia-self and a willingness to make changes in life to facilitate and support healthy sleep without expectations. This acceptance includes acknowledgement that some nights will be better than others, but regardless, the next day will still come, they will function reasonably well, and the coming night offers another opportunity for a better night’s sleep. This humility attenuates death anxiety and insomnia, allowing the mind to wander into more pleasant pastures of thought: the kind that beckons sleep rather than chasing it away. Maybe existentialism is what we need to help people sleep. The thought that the universe is absurd, and therefore, nothing really matters, including whether or not you sleep tonight.

## Figures and Tables

**Figure 1:**
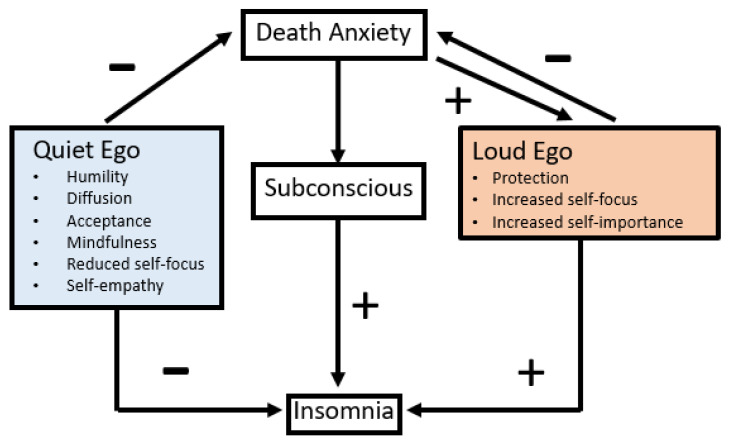
Theoretical model of how death anxiety may foster development of insomnia. The impact of death anxiety on the subconscious likely fosters insomnia. Death anxiety fosters loud ego orientations. Both the quiet and loud ego can allay death anxiety. However, quieting the ego results in positive cognitive behavioral effects such as mindfulness, humility, reduced self-focus and acceptance that can mitigate or hinder insomnia. Loud ego protectionism, increased self-focus, and increased self-importance can mitigate death anxiety but also trigger or foster insomnia. Each ego orientation (e.g., loud or quiet) can undermine the psychological protection afforded by the other. Thus, the specific ego orientation used to address death anxiety may either hinder or foster insomnia development (note: arrows with “+” labels foster the behavior they point toward, while arrows with a “−“ label hinder the behavior they point toward).
